# Comparison of High and Low Responders to a Cross-Country Skiing Talent Transfer Program: A Coach’s Perspective

**DOI:** 10.3390/sports9100138

**Published:** 2021-10-02

**Authors:** Stig Arve Sæther, Mats Iversen, Rune Kjøsen Talsnes, Øyvind Sandbakk

**Affiliations:** 1 Department of Sociology and Political Science, Norwegian University of Science and Technology, 7491 Trondheim, Norway; mats.iversen@hotmail.com; 2 Meråker High School, Trøndelag County Council, 7735 Steinkjer, Norway; runta@trondelagfylke.no; 3 Department of Sports Science and Physical Education, Nord University, 8049 Bodø, Norway; 4 Centre for Elite Sports Research, Department of Neuromedicine and Movement Science, Norwegian, University of Science and Technology, 7491 Trondheim, Norway; oyvind.sandbakk@ntnu.no

**Keywords:** endurance sport, performance development, cross-country skiing, talent transfer, training responses, Winter Olympic Games

## Abstract

Purpose: To examine how coaches differentiate athletes with successful and non-successful development during a cross-country (XC) skiing talent transfer (TT) program. Methods: We conducted qualitative, semi-structured interviews with seven Norwegian coaches working with a group of 23 Chinese summer endurance athletes transferring from running, rowing, and kayaking to the winter endurance sport XC skiing over a six-month training period. The athletes were grouped as either high (*n* = 9), moderate (*n* = 3), or low responders (*n* = 11) based on objective performance development, quantified using laboratory testing. The interview guide contained six sections: physiological development, technical development, psychological characteristics, training and recovery routines, athlete background, and considerations about the effectiveness of TT initiatives in general. Results: The assessments of the coaches revealed that greater development of both physiological and technical capacities among the high-responding TT athletes were associated with higher motivation, as well as superior ability to deal with adversity in the development process. Conclusion: The coaches considered the TT program to be effective; however, successful transfer of athletes to a world class level in a complex sport such as XC skiing requires a multidisciplinary selection process and a longer time frame than the six-month period used in the current project.

## 1. Introduction

For several decades, coaches, athletes, and researchers have searched for keys to optimize performance development among elite athletes [1]. Most of the previous literature has focused on the development process of athletes within a specific sport [2]. However, an alternative approach is talent transfer (TT, also referred to as athlete transfer). TT can be defined as the process in which athletes make a change from their original sport (i.e., donor sport) to a new sport (i.e., transfer sport). TT has been adopted by various sport government bodies as a means of capitalizing on the developmental investment made in previously identified athletes (‘recycling’) and fast-tracking athletes to new sports where they might have a greater chance to succeed [3]. The overall idea of TT initiatives is to recruit athletes who have shown a high level of performance in a sport, through investment in both time and effort, without taking the last step to an elite level, and they are therefore provided the opportunity to transfer to a new sport where they might have better potential for success. These programs have been conducted in various sports during the last several decades, despite claims of low success rates and a high degree of serendipity, such as in Great Britain prior to the London Olympics in 2012 [4]. The TT approach has mainly been used in summer sports, but the latest approach by China is directed towards select winter sports, aiming to develop athletes for the upcoming Olympic Games in Beijing 2022 (https://www.chinadaily.com.cn/china/2017-08/08/content_30364992.htm, accessed on 11 August 2021). Research has found a close relationship between the host country and the number of medals won in the competitions [5]. The Olympics has become a showcase, making the host country want to invest in their own games to win the overall medal count in their host Olympics. To be able to be among the most successful nations in the medal count, the host nations are dependent on developing athletes that can compete at an international level in most sports held during the Olympics. Investing in a TT program could increase the possibility to perform in sports that the nations, historically, have been less successful in. Still, a recent systematic review showed that the available evidence on TT is scarce and included only five studies, and it concluded that similarities between donor and transfer sports are associated with success and that psychological factors play an important role for individual athletes’ development in the TT program [6]. Furthermore, most studies, both on talent development and TT, have been conducted on western athletes, even though some recent examples of studies are done among Chinese athletes [7].

One vital aspect of TT programs is to understand the connection between ‘donor’ sports (the original sport an athlete used to practice in) and ‘recipient’ sports (the destination sport to which an athlete has transferred) [8]. Currently, the connection between ‘donor’ sports and ‘recipient’ sports is less examined; although, Talsnes et al. [9] found runners to improve more than kayakers and rowers when Chinese athletes were transferred to cross-country (XC) skiing over a six-month training period. Another important aspect is to find the appropriate selection criteria, in which previous studies have criticized TT programs for adopting inappropriately narrow criteria, focusing mainly on physical and anthropometric factors [10]. Accordingly, studies have emphasized the importance of assessing athletes’ abilities to learn in new, knowledge-rich environments. Furthermore, the role of psycho-behavioral factors and self-regulation to foster the use of knowledge and skills that should facilitate effective transfer has been emphasized [11,12]. In other words, the authors argue that a greater effort should be placed on identifying an athlete’s capacity to learn instead of measuring what has already been learned.

In an approach by Talsnes et al. [9], a group of 24 Chinese runners, rowers, and kayakers were transferred to XC skiing, one of the most technically and physiologically demanding endurance sports, over a six-month training period using state of-the-art training methodology [13] and high-level coaches. Here, large improvements in the development of sport-specific capacities (i.e., roller skiing and double-poling ergometry) were measured in the laboratory, with fewer changes in more general capacities (i.e., running) [9]. In a follow-up study by Talsnes et al. [14], they compared high and low responders in the same group of TT athletes, and better physiological adaptations were found among high responders, whereas technical capacities were largely improved in both groups. To separate the athletes that best responded to the six-month training period, a quantitative performance index was used to classify high- and low-responders. Relative changes from pre to post peak velocity during an incremental treadmill running and roller-ski skating test, in addition to average power output during both 30 sec and 5 min double-poling ergometry tests, were used to determine this index. Superior physiological improvements coincided with a higher training load, higher training volume, and less injury and illness during the training period in the group of high responders [14]. However, the underlying mechanisms related to the ability to concurrently respond both physiologically and technically, as well as the factors associated with high training tolerance and low injury rates, are not known. Here, the study by Talsnes et al. [14] indicated that high motivation and strong coach–athlete alliances played a significant role, which are aspects that require more attention in future studies.

While the selection and identification process related to talent development is widely investigated (for a review, see Till et al. [1]), the TT process could be considered an exploration of the rest of the potential from athletes that have achieved a high-performance level without reaching a world-class level within their original sport. The idea of doing a TT is to use the athlete’s potential in a donor sport and invest training in a ‘recipient’ sport, where the athlete is regarded to have the potential to reach a high international level. The athlete’s level of progress (response) is therefore vital in the TT process. Building upon the previous study by Talsnes et al. [14], the primary aim of the present study was, therefore, to examine how coaches differentiated high- from low-responding athletes in a six-month XC skiing TT program. Since both the TT process and identification of athletes have been studied to a lesser degree, a secondary aim was to examine how coaches perceived the value of such TT initiatives based on their experience as elite coaches.

## 2. Materials and Methods

In this study, a qualitative approach was chosen to gather detailed information from Norwegian XC-skiing coaches working with TT athletes about their reflections on differences between successful and less successful development in their respective TT program, used to develop XC skiers aiming for success in the Beijing 2022 Winter Olympics. Laboratory tests were performed to determine the TT athletes’ development in performance, physiological, and technical capacities as described in detail by Talsnes et al. [9]. These data further defined the groups of responders and non-responders and thereby formed the basis for the qualitative interviews presented in this study. The coaches were aware of these results when they were interviewed regarding the TT athletes.

### 2.1. Participants

The seven interviewed coaches (six men, one woman) were all experienced Norwegian XC-skiing coaches who worked on the project during the entire six-month period (see Table 1). The age and years of coaching experience for the group was (mean ± standard deviation [SD]) 29 ± 4 and 6 ± 5 years, respectively. The group consisted of some younger coaches with 1–2 years of coaching experience and some older coaches with 7–15 years of experience, including one previous national team head coach in XC skiing for Norway and one previous head coach for the development team in the biathlon for Norway. The project was conducted in Meråker, Norway from November 2018 to May 2019. All participants volunteered for this study and signed informed consent forms prior to their participation. The research was undertaken according to the ethical guidelines of the Norwegian Centre for Research Data (NSD).

### 2.2. Data Collection

Prior to the interview, participants were informed both verbally and in writing about the study and interview. During the interviews, a semi-structured approach was adopted where an identical set of questions was employed in a similar manner. Although this procedure resulted in a certain element of structure to each interview, the ordering of questions varied depending on the responses of each participant, where some of the issues raised were explored further by the interviewer. Although the discussions varied in their content due to the participant responses, a variety of probe (e.g., ‘Why do you think this is?’) and elaboration (e.g., ‘What do you mean by that?’) questions were employed to ensure that all issues were investigated in depth (see Table 2). At the end of all interviews, participants were asked if all appropriate factors had been discussed and if they wanted to add anything regarding some of the aspects, and none of them did. All of the interviews were conducted face-to-face in an environment comfortable for the participant, and the interviews were also tape-recorded in their entirety (30–45 min duration) and transcribed verbatim.

### 2.3. Data Analysis

The interviews were audio recorded and transcribed. All players were given an identification code, C1–C7 (Coach 1–Coach 7), to provide anonymity. According to the six steps suggested by Braun et al. [15] for qualitative analysis, the data were analyzed using the following process: (1) transcribing, reading, and re-reading the data; (2) generating initial codes, such as physiological and technical development; (3) applying deductive codes and identifying lower-order themes, such as motivation within psychological characteristics; (4) outlining of the overall topics from the data, such as training and recovery routines; (5) reviewing and refining the subthemes and final categories; (6) writing a report and presenting the data (p. 191). Having gone through all the data from the interviews, we ended up with our six topics presented in the results section.

In this manuscript, rigor is considered through the meaningful coherence between the purpose of the study, the procedure, and the findings [16]. Building on Tracy [16], we have sought to ensure transparency by making a detailed description of the research process and role by creating distance when reflecting and interpretating our findings. Moreover, we have continually sought to verify and validate the analysis and provide critical interpretations of the data. During the data collection, the research team discussed various results thereby ensuring peer validity [17]. Additionally, ongoing member reflections took place during the individual studies [16], with the intention of making sure that the descriptions and explanations were rich, bountifully supplied, generous, and unstinting.

### 2.4. Performance Index

The athletes’ development of performance, physiological, and technical capacities were measured in the laboratory as described in detail by Talsnes et al. [9]. A performance index was developed to classify high and low responders to the six-month training period [14]. Pre- to post-relative changes in performance measures during incremental running and roller-ski skating tests, in addition to average power output in a 30 s and 5 min double-poling ergometry test, were used. These four performance measures were further summated, resulting in the performance index with cutoffs set to classify high (*n* = 9), moderate (*n* = 3), and low responders (*n* = 11). Moderate responders were excluded from further analyses to establish distinct group differences. High responders consisted of eight men and one woman, whereas low responders consisted of six men and five women, respectively. Furthermore, eight athletes in the high responder group and five athletes in the low responder group had a previous sports background in running (i.e., long or middle distance), whereas the remaining athletes in both groups had a sports background in kayaking or rowing.

## 3. Results

The current investigation examined how coaches differentiated athletes with successful and non-successful development in a XC skiing TT program. These are presented for each of the interview topics below (see Table 3).

### 3.1. Physiological Development

In general, most coaches (5) stated that the greatest development was seen in sport-specific physiological capacities (i.e., roller-ski skating and double-poling ergometry), whereas more general physiological capacities (i.e., running) were less changed. Regarding the development of general capacities, there were different opinions between the coaches. Although, most of the coaches (4) that perceived those general physiological capacities seemed unchanged in the entire group, one of the coaches reported that the athletes who responded positively to the training developed both sport-specific and general physiological capacities:

C2: ‘The ones who have a positive development, have development in all capacities I would say. Even general physiological capacities.’

One coach points out that the development of physiological capacities for the athletes varied, and some of them had good development also in this aspect:

C4: ‘For some, the development of physiological capacities has been good. Others had almost no development, while some had negative development of their physiological capacities.’

Most of the coaches (5) experienced that the highest responders had good development in maximal oxygen uptake (VO_2max_), while the lowest responders had little or even negative changes.

In addition, a high initial aerobic endurance capacity is highlighted by several coaches (3) as one of the most important characteristics of high responders. In particular, the high endurance capacity of the young runners, especially the men, seems to be a determining factor for a successful transfer to XC skiing, and several of the coaches (2) point this out in the following quotes:

C6: ‘It seems that athletes transferring from running improved more than athletes transferring from kayaking and rowing. Also, the boys revealed better development than the girls, together with athletes with high initial endurance capacities. The youngest athletes had also better physiological and technical development than the older ones.’

C7: ‘It’s the former runners who developed the most…They had a good endurance capacity when entering the project, and you may think that running is more comparable to the technical patterns in XC skiing than rowing or kayaking. Thus, runners had endurance capacities and muscular characteristics more relevant for the demands of XC skiing.’

While all of the coaches agreed that strength and endurance capacity is important in XC skiing, they also highlight the importance of a good perception of the technical aspects of XC skiing as crucial:

C4: ‘Several of the athletes had better physiological capacities than their Norwegian peers, but this does not reflect their actual performance in competitions. It’s the ski perception of skiing that matters the most and how to efficiently go on skis. They still miss this feeling.’

C3: ‘You have to obtain a minimum level of VO_2max_ but still, it is not necessarily the one with highest VO_2max_ who is the best performing skier.’

### 3.2. Technical Development

Three of the coaches emphasized technical development as the most crucial factor for performance development, as outlined in the following quote from a coach:

C5: ‘It’s clear that technical development is the most important factor. The ones who perform best in competitions are the ones in the program who have had largest technical development.’

The coaches seem to have different opinions on the main factors associated with technical development. Some coaches (2) consider the ability to positively respond to feedback as the most important factor of technical development, and they highlight the importance of a strong coach–athlete relationship in this context. They state that the ones with the largest technical development are the ones who are constantly seeking technical feedback from their coaches:

C6: ‘The ability to positively respond to feedback, so they can work with the things they are instructed to the highest responders understand they have to work with the technical tasks provided, not only for that specific session, but over a long period of time, even the times when there is no one watching them’.

The coaches seem convinced that concurrent development of both physiological and technical capacities is crucial for performance development in XC skiing. However, due to the large demand for rapid technical development in athletes transferring from summer sports to XC skiing, the coaches argue that more emphasis should be placed on the athletes’ technical capacity, to learn technique, enjoy skiing, respond well to feedback, and develop motivation to work with technical tasks over time.

### 3.3. Psychological Characteristics

All of the coaches point out motivation as crucial for performance development in the process of transferring to XC skiing, and many coaches (3) also highlight the athlete’s curiosity and desire to develop as important for inducing positive training responses. As one of the coaches says:

C1: ‘Motivation, enjoyment and passion. Curiosity and the desire to develop and become better. These are clearly characteristics of the highest responders if you are not happy and motivated with what you are doing in life, then it doesn’t matter what you do. If you have the best coaches, the best training program, it doesn’t matter If they don’t like what they are doing, then it doesn’t work.’

Others (2) emphasize the ability to deal with adversity, as quoted below:

C7: ‘Maybe the lowest responders give up easier due to lack of inner passion to improve.’

C6: ‘The best athletes give it several tries before they give up; they react constructively to feedback, and don’t give up and this motivation can be hard to maintain in this project. That’s why the athlete’s wellbeing is highly important. To keep up their motivation.’

When asked about characteristics of the highest responding athletes, the coaches showed many similarities in their opinions, with high motivation as a main point described from the following statements:

C6: ‘It’s the ones who have been most motivated. The most motivated athletes do as they are told, and you see they are more focused and have more discipline. They are ready for training sessions early, are concentrated during the sessions, and you see that they want to become better athletes.’

C4: ‘It’s the ones who have maintained the highest motivation, have been most satisfied with living here in Norway, and have established a good environment and training group.’

In addition to a high motivation, other mental capacities associated with a large development in XC skiing were outlined, such as self-criticisms, reflections, and wellbeing:

C6: ‘The ones who are critical to themselves and to the training program we carry out develop well. The best have always asked a lot of questions, reflect if they do things good enough, and are constantly looking after better ways to develop the ones who doesn’t [sic] try and fail hard, have stagnated.’

C1: ‘They see the joy in training, like the new life situation here. And their wellbeing.’

### 3.4. Training and Recovery Routines

The coaches also highlighted the importance of optimizing the balance between training and recovery, of individual adjustments of training load, and of the absence of injuries and sickness. Some of the coaches (3) perceived athletes with the greatest performance development as the ones who were more able to adjust their training load. Especially early in the project, one coach felt that the best responders were the ones who made more adjustments in their training:

C5: ‘Some of the athletes were better than others at making small adjustments in their training load. These ones probably perceived the balance between load and recovery better, and therefore had a more positive performance development.’

Other coaches (2) perceived that there was no clear relationship between the athlete’s ability to adjust their training load and their subsequent training response. Here, most athletes completed the same training sessions and thus had a similar overall training load; although, each athlete’s training response differed extensively. As coach 3 mentions:

C3: ‘I see no clear relationships between making adjustments in load and performance development among these athletes. I feel that some of the ones who have responded negatively to the training also have tried to do everything right, including adjustments of load, but just without having the same training response and performance development as others.’

Most of the coaches (5) point out that this aspect has been a challenge during the TT program since most athletes were not familiar with making individual adjustments in their daily training. One coach also felt this had been a challenging aspect of the entire TT project:

C5: ‘Either it’s us [sic] who hasn’t [sic] communicated this well enough, or it’s cultural differences between Norway and China in the training and development of endurance athletes which explains this challenge.’

One of the coaches felt that it was the athletes who performed ‘extra sessions’ who are the highest responders, as pointed out in the following quote:

C2: ‘It’s like the ones who become the best are the ones you often see out on an extra training session, the ones who dare to train a little more than the others.’

However, coach 7 has a different opinion on this:

C7: ‘On rest days, some of the athletes performed individual training sessions. These ones are not the highest responding athletes, but the ones to strive to catch up with the rest the best ones are normally better at recovery so it’s often the ones who are a little behind and think they have to do some extra training between the sessions. But these ones are not the best.’

One other factor that is pointed out as important for the athletes’ development is continuity in their training process (4). Injuries and illness can negatively influence adaptations and performance development, and the highest responders seem to have more continuity in their training and less injuries:

C3: ‘It’s the athletes who have continuity in their training, stay healthy and free of injury in addition to finding the optimal balance between load and recovery which had the largest performance development…’

C1: ‘Yes, you can see that it’s the ones in the lowest responding group who have most cases of sick days and days with injuries, that is clearly.’

One of the coaches had another take on this and claimed that the athletes who challenged themselves the most also had more injury days due to falling on the slopes:

C2: ‘but the ones with best development are also the ones that dare to challenge themselves, and due to this, the highest responding group has some more days with injuries as a result of falling on roller-skis/skis.’

### 3.5. Athlete Background

When asked about the athletes’ sports background and how this may have influenced differences in performance development between athletes, all coaches shared the same opinion that runners most positively responded to a XC skiing TT initiative:

C3: ‘The rowers seem to have “heavy legs” and poor development; the kayakers seem to fit a little better, and have somewhat better development, but it’s definitely the runners who developed the most during the six-month training period.’

All of the coaches seemed to agree that many of the oldest athletes struggled to develop, especially technically, and seemed to have a harder time adapting new movement patterns, as coach 7 pointed out:

C7: ‘It’s like the older ones are harder to adapt to a new movement pattern. So, it seems to be preferable with the younger athletes. And also, mentally, the younger ones seem to be more curious and unexperienced’.

In addition, some of the coaches (2) point out that athletes from rowing and kayaking had more muscles and higher body mass than the runners, which could be a disadvantage for developing as skiers. As coach 5 says:

C5: ‘You have to carry your own body weight when you are skiing, and some of them seemed untrained they were a little bit too big, strong in the upper body, but weak in the core and the lower parts of the body.’

Furthermore, it seems to be an agreement among the coaches that the men displayed better development than the women. As the coaches highlight:

C2: ‘It can be the differences in gender, in how we are built. It can be the amount of testosterone in the boys, it can be the toughness within the boys because of the testosterone, and it can be that the girls perform better at lower intensity but struggle to develop high top speed.’

C5: ‘It seemed like the boys liked it better here. They were like a boy’s club who were at a training camp here and had an awesome time together with each other both during and between the training.’

The last quote is related to the athlete’s motivation, which was regarded as one of the most important characteristics of high responders.

### 3.6. Considerations about the Effectiveness of Talent Transfer Programs

All coaches pointed out that the athletes’ development during the six-month period had been impressive. However, the development was not linear and leveled out during the last weeks of the project, as coach 4 points out:

C4: ‘Isolated, they have developed a lot. In the beginning, the progression curve was steep, while now, we are finding us in a period where we have somewhat less progress in performance.’

Coach 1 points out that even though he is satisfied with the development they have seen, his satisfaction is limited because of the ambitious goal of the project:

C1: ‘On one side, I am very satisfied with the development the athletes have had on skis. In a 10 km race, they came 30 min behind their Norwegian peers in the beginning, while they are now only 2–5 min behind the same athletes today. But on the other side, when the project has the 2022 Olympics as a goal, the athletes’ performance is far from a level good enough for World Cup and Olympic races.’

The coaches’ impression of TT initiatives as a development model seemed to differ, but all of the coaches pointed out many positive aspects of the TT model, and it seemed to work pretty well, with some precautions. One coach pointed this out in the following quote:

C2: ‘It works well if you start with 2000 athletes you select from, and always narrow it in I’m sure that when you come down to 10–20 highly motivated athletes, who work hard and like to train, and if you work with these ones over a long period, maybe 6 to 10 years, then I am 100% sure you would get results in XC skiing.’

Some of the coaches (3) pointed out that they think it is a good model for development of sporting talents, but it can be a challenging approach in XC skiing because of the complex demands of the sport. Coach 1 outlines this in the following quote:

C1: ‘The project has worked well but regarding TT and XC skiing, it’s something I wouldn’t recommend. I think it is an advantage to have more “ski feeling” from early stages in life.’

This is something another coach also points out:

C6: ‘They have absolutely no history with XC skiing from before, and they started pretty late I think the transfer model would work better in some other endurance sports, because XC skiing has so many technical aspects, so to perform well is hard I think it would be easier to transfer their physical capacities to for example cycling or rowing.’

When the coaches were asked about what changes they would have made if they could have started the project over again, they have different answers. Three of the coaches pointed out that the selection process was not optimally handled, as highlighted in the following:

C7: ‘I would have spent more time with the selection process, had more specific tests and conducted personal interviews with each athlete, to hear their thoughts about the project and how their motivation was.’

Two of the coaches also highlighted more playing and less seriousness in the beginning of the project as something they would have changed in retrospect. This would have made the skiing-specific technical aspects easier to manage for the athletes. One of the coaches pointed this out in the following quote:

C4: ‘A little more playing on skis in the beginning. Even more than we did. In the beginning this was forgotten, and things got a little too serious in retrospect this would have helped them become better in downhills.’

Another aspect pointed out is that the ‘donor athletes’ should have had more experience with XC skiing when arriving. This would have made the technical development easier and would have made the progression faster. One coach said it as:

C6: ‘If you had taken a group that had tried skis before, maybe a group of runners for example with some kind of experience with skiing then I would assume they would have had better development.’

This section may be divided by subheadings. It should provide a concise and precise description of the experimental results, their interpretation, as well as the experimental conclusions that can be drawn.

## 4. Discussion

The present study examined how coaches differentiated athletes with successful and non-successful development during a six-month XC skiing TT initiative. The main findings were as follows: (1) The coaches experienced that a greater development of both physiological and technical abilities in high responders was coupled with a superior motivation for learning a new sport and the development process, which led to higher performance development in XC skiing compared to low responders. (2) Athletes transferring from running, especially men, adapted more rapidly to XC skiing and tolerated XC skiing-specific training better. (3) The coaches were more ambiguous about the importance of the ability to perceive and adjust training load or to deal with adversity in the development process for differentiating high responders from lower-responding peers. (4) The coaches considered the TT program to have potential for athlete development, although the TT program seemed less appropriate for such a complex and technically demanding sport as XC skiing.

As expected, all athletes improved their sport-specific performance following six months of XC ski-specific training. However, as previously described by Talsnes et al. [14], the coaches perceived that the highest-responding athletes concurrently improved physiological capacities in both general (i.e., running) and sport-specific (i.e., roller-ski skating and double-poling ergometry) exercise modes. Here, the coaches emphasized that high responders especially developed their XC skiing technique more rapidly due to a higher motivation for learning a new sport and for the development process in general leading to a larger performance development in XC skiing, in comparison to the low responders. This consequently influenced the ability to work hard with given technical assignments, that involved searching for individual solutions in addition to a superior ability to react to feedback from coaches. A vital challenge for most athletes was, however, the lack of perception for the technical aspects related to skiing, which, according to the coaches, limited their ability to develop as XC skiers.

In this context, the athletes’ age and gender seemed to have influenced their technical development, as some coaches stated that the youngest male athletes in the project developed their technical capacities the most. The fact that the youngest athletes demonstrated better technical development than the older ones corresponds with previous literature on TT initiatives [8]. Furthermore, the fact that athletes with backgrounds in running were most represented in the high-responder group is further supported by the findings of Talsnes et al. [9], which found a significantly larger sport-specific performance development among runners than kayakers and rowers. Furthermore, this also agrees with Vaeynes et al. [8], who pointed out that some sports are more likely to act as donor sports in TT initiatives, and additionally, they outlined rowing and kayaking as sports that may be more suitable as recipient sports than donor sports. Furthermore, anthropometric factors, such as body height and weight, were highlighted by the coaches as important factors, as runners with lower body weight developed more than rowers and kayakers with higher body weights, which also agrees with the findings of Rees et al. [18]. Overall, the most successful athletes of the project were able to concurrently develop their physiological and technical capacities, which coincided with a high level of motivation for the development process, and thereby an improved working alliance with their coach.

These findings are supported by previous literature, emphasizing psychological factors as a main determinant of successful performance development in sports [18,19]. As mentioned above, the coaches particularly pointed out motivation as the most important factor associated with a successful transfer from summer sports to XC skiing over a six-month training period. Here, the coaches related high motivation to have an influence on several aspects among the high responders, such as a superior ability to reflect upon their own training process and to deal with adversity. The coaches were, however, more ambiguous on how the ability to reflect upon the training process and to deal with adversity was different between the two groups of athletes. This might be explained by the normally high training load expected by XC skiers [9]. Altogether, these findings are supported by the existing literature, which highlights motivation as a key factor with influence on several underlying mechanisms related to sports development [12,19,20]. 

When we asked the coaches how they considered this TT initiative as a model for developing world-class XC skiers, the coaches had two-sided opinions on its effectiveness. On one hand, the coaches were satisfied with the large progress of the athletes during the six-month training period in Norway. On the other hand, all of them pointed out several challenges with the ambitious goal of the project, developing XC skiers in such a short period. Thus, XC skiing takes, most likely, longer time to learn and may therefore not be the optimal sport for TT initiatives. In addition, some of the large differences in development between athletes may have been caused by the selection process of the current TT program. Thus, this process should have been performed in a more sophisticated manner, involving experts in XC skiing, which could have resulted in fewer low responders, due to a selection of younger athletes with a larger physical (including sports background) and motivational potential for a transfer to XC skiing.

Seen in retrospect, the coaches stated that it would have been preferable if the athletes had more experience with XC skiing before they came to Norway and started the TT process. Lastly, the coaches would have put more focus on play and less on seriousness in the initial phase of the project. Such aspects were suggested to have a positive influence on the technical development in the later phases of the project, which was considered the most crucial skill to develop within the project. Here, the coaches’ assumptions corresponded well with previous findings on TT initiatives [8,19], as a more thorough selection process by recruiting athletes from sports with similar physical characteristics, such as the coaches highlighted, could have made the program more efficient in the goal of developing high-level XC skiers.

### 4.1. Methodological Considerations

The present study extended upon previous findings by Talsnes et al. [14], using a qualitative research design to provide a more holistic understanding of the underlying mechanisms explaining successful versus less successful TT development to XC skiing. We believe that the main strength of the study is the comprehensive amount of data. This is supported by Martindale et al. [20] who argued that an athletic development process should be considered in a holistic manner to gain insight into what an effective talent development environment (TDE) is. The main limitation is related to cultural differences and the language barrier since these aspects could lead to wrong interpretations by the coaches. Another obvious limitation is the lack of athletes’ perspective on the TT process in our design, which most likely would have given complementary insights into the mechanisms explaining successful versus less successful TT to XC skiing.

### 4.2. Practical Implications

Conducting a TT initiative has obvious challenges for an athlete, which is particularly related to changing from a well-known sport to a less known sport and to trying to reach an elite level over a relatively short time. As earlier research has highlighted [6], such transfer initiatives would be easier when there are large similarities between the donor and the transfer sport, indicating that both athletes and coaches should highlight the importance of choosing a relevant transfer sport. Still, the same research has shown that psychological factors play an important role in TT with the high quality required associated with effective talent development. Our study confirmed the earlier studies on both these topics, indicating that there are donor sports that seemed more physiologically suitable for recruitment to XC skiing, even though the athletes’ psychological skills were considered additionally essential by the coaches. Overall, the coaches in this study believed in the general concept of TT, but they highlighted that the identification process must be performed more thoroughly and should be based on experts in the transfer sport. These results would indicate that TT is an aspect to consider for most sport governing bodies to supplement their talent development process. Still, the coaching TT athletes would probably require different approaches compared to their “normally recruited” athletes.

## 5. Conclusions

The assessments of coaches working with a group of summer endurance athletes transferring to XC skiing revealed that greater development of both physiological and technical capacities among the high-responding TT athletes were associated with higher motivation, greater ability to perceive and adjust their training, as well as superior ability to deal with adversity in the development process. More specifically, superior motivation for learning a new sport and the development process leading to a higher performance in XC skiing seemed to be key differences between high and low responders. Most of these high responders, who also adapted faster to XC skiing and tolerated the training better than low responders, had a background in running and were younger men. Furthermore, according to some of the coaches, the better ability to perceive and adjust training load appropriately might have induced higher compliance to training, and thereby larger training loads as well as fewer injuries and less sickness, even though other coaches did not share this opinion. The coaches agreed that superior ability to reflect upon their own training process and deal with adversity differentiated high responders from their lower-responding peers. In general, the coaches considered the TT program to be positive; however, successfully transferring athletes to a world class level in a complex sport such as XC skiing requires a multidisciplinary selection process and a longer time frame than the six-month period used in the current project.

## Figures and Tables

**Table 1:** Coach demographic information.

Coach	Gender of Coach	Sport	Gender of Athletes	Coach Experience
Coach 1	Male	Cross-country skiing	Male/Female	Medium 3–6 years, regional/national
Coach 2	Male	Cross-country skiing	Male/Female	Highly 6+ years, elite/international
Coach 3	Male	Cross-country skiing	Male/Female	Little 1–3 years, local/club
Coach 4	Male	Cross-country skiing	Male/Female	Little 1–3 years, local/club
Coach 5	Male	Cross-country skiing	Male/Female	Medium 3–6 years, regional/national
Coach 6	Male	Cross-country skiing	Male/Female	Little 1–3 years, local/club
Coach 7	Female	Cross-country skiing	Male/Female	Little 1–3 years, local/club

**Table 2:** Examples of interview questions.

Physiological development: How is the correspondence between the development in cross-country skiing performance and the physiological development of the athletes?
2. Technical development: How crucial is the technical development in cross-country skiing for performance?
3. Psychological characteristics: What mental qualities characterize the skiers with best performance-development?
4. Training and recovery routines: How do athletes balance training and recovery?
5. Athlete background: What would you say are the sports backgrounds of those with the greatest performance-progress?
6. Considerations about the effectiveness of TT initiatives: What do you consider as the success factors for a successful TT?

**Table 3:** Emerging themes and sub-categories following the process of thematic analysis.

Physiological Development	Technical Development	Psychological Characteristics	Training and Recovery Routines	Athlete Background	Effectiveness of Talent Transfer Programs
-Sport-specific physiological capacities (5)	-Most crucial factor for performance development (3)	-Motivation (7)	-Balance optimizing (7)	-Runners positively responders (7)	-Short-term progress (7)
-Lack of development in general physiological capacities (5)	-Positive response to feedback (2)	-Curiosity and desire (3)	-Training load adjustment (3)	-Younger athletes positively responders (7)	-Positive impressions (7)
-Training developed both sport-specific and general physiological capacities (1)	-Combination of physiological and technical capacities (7)	-Dealing with adversity (2)	-Lack of ability to adjust training load and training response (2)	-Rowers and kayakers’ disadvantage (2)	-Positive development, less in XC skiing (3)
-Variation in development of physiological capacities (1)		-Self-criticisms, reflections, and wellbeing (5)	-Adjustment of training load challenge during the TT program (5)	-Men positive responders (7)	-More specific selection criteria (3)
-Maximal oxygen uptake (5)			-‘Extra sessions’ characterized highest responders (1)		-More play and less seriousness in the beginning (2)
-Initial aerobic endurance capacity (3)			-Catch up training not the way to go (1)		-‘Donor athletes’ with experience with XC skiing (5)
-Endurance capacity of young male runners (2)			-Injuries and illness (4)		
			-Self-challenging athletes more injury days (1)		

Numbers in parentheses represent number of coaches highlighting each themes/sub-category.

## Data Availability

The data are not publicly available as data sharing was not included within the original study ethics submission or participant consent form.

## References

[1] Till K., Baker J. (2020). Challenges and [Possible] Solutions to optimizing talent identification and development in sport. Front. Psychol..

[2] Bridge M.W., Toms M.R. (2013). The specialising or sampling debate: A retrospective analysis of adolescent sports participation in the UK. J. Sports Sci..

[3] Halson S., Martin D.T., Gardner A.S., Fallon K., Gulbin J. (2006). Persistent fatigue in a female sprint cyclist after a talent-transfer initiative. Int. J. Sports Physiol. Perform..

[4] Collins R., Collins D., MacNamara Á., Jones M.I. (2014). Change of plans: An evaluation of the effectiveness and underlying mechanisms of successful talent transfer. J. Sports Sci..

[5] Weber A.C., De Bosscher V., Kempf H. (2018). Positioning in Olympic Winter sports: Analysing national prioritisation of funding and success in eight nations. Eur. Sport Manag. Q..

[6] van Harten K., Bool K., van Vlijmen J., Elferink-Gemser M. (2021). Talent transfer: A systematic review. Curr. Issues Sport Sci..

[7] Bonal J., Jiménez S.L., Lorenzo A. (2020). The Talent Development Pathway for Elite Basketball Players in China. Int. J. Environ. Res. Public Health.

[8] Vaeyens R., Güllich A., Warr C.R., Philippaerts R. (2009). Talent identification and promotion programmes of Olympic athletes. J. Sports Sci..

[9] Talsnes R.K., Hetland T.-A., Cai X., Sandbakk Ø. (2020). Development of performance, physiological and technical capacities during a six-month cross-country skiing talent transfer program in endurance athletes. Front. Sports Act. Living.

[10] MacNamara Á., Button A., Collins D. (2010). The role of psychological characteristics in facilitating the pathway to elite performance part 2: Examining environmental and stage-related differences in skills and behaviors. Sport Psychol..

[11] Bransford J., Schwartz D. (1999). Rethinking Transfer: A Simple Proposal with Multiple Implications.

[12] MacNamara Á., Button A., Collins D. (2010). The role of psychological characteristics in facilitating the pathway to elite performance part 1: Identifying mental skills and behaviors. Sport Psychol..

[13] Sandbakk Ø., Holmberg H.C. (2017). Physiological capacity and training routines of elite cross-country skiers: Approaching the upper limits of human endurance. Int. J. Sports Physiol. Perform..

[14] Talsnes R.K., van den Tillaar R., Cai X., Sandbakk Ø. (2020). Comparison of high- vs. low-responders following a 6-month XC ski-specific training period: A multidisciplinary approach. Front. Sports Act. Living.

[15] Braun V., Clarke V., Wheate P., Smith B., Sparkes A. (2016). Using thematic analysis in sport and exercise research. Routledge Handbook of Qualitative Research in Sport and Exercise.

[16] Tracy S.J. (2010). Qualitative quality: Eight ‘big-tent’ criteria for excellent qualitative research. Qual. Inq..

[17] Kvale S., Brinkmann S. (2009). Interviews: Learning the Craft of Qualitative Research Interviewing.

[18] Rees T., Hardy L., Güllich A., Abernethy B., Côté J., Woodman T., Montgomery H., Laing S., Warr C. (2016). The great British medalists project: A review of current knowledge on the development of the world’s best sporting talent. Sports Med..

[19] McCormick A., Meijen C., Marcora S. (2015). Psychological determinants of whole-body endurance performance. Sports Med..

[20] Martindale R.J., Collins D., Daubney J. (2005). Talent development: A guide for practice and research within sport. Quest.

